# Atomic simulation of crystal orientation and workpiece composition effect on nano-scratching of SiGe alloy

**DOI:** 10.1186/s11671-023-03859-9

**Published:** 2023-06-26

**Authors:** Changlin Liu, Suet To, Xuexiang Sheng, Ruoxin Wang, Jianfeng Xu

**Affiliations:** 1 State Key Laboratory of Ultra-precision Machining Technology, Department of Industrial and Systems Engineering, The Hong Kong Polytechnic University, Kowloon, Hong Kong China; 2 State Key Laboratory of Intelligent Manufacturing Equipment and Technology, School of Mechanical Science and Engineering, Huazhong University of Science and Technology, Wuhan, China; 3 Shandong Harbour Engineering Group Co., Ltd, Shandong Port Group, Rizhao, China; 4 The Hong Kong Polytechnic University Shenzhen Research Institute, Shenzhen, China

**Keywords:** Silicon–germanium alloy, Molecular dynamics simulation, Nano-scratching process, Subsurface damage

## Abstract

Silicon–germanium (SiGe) alloy is a new semiconductor material of great interest in thermoelectric devices, optoelectronic devices, infrared detectors, and semiconductor industry. In the present work, molecular dynamics simulation was conducted to investigate the deformation behavior in nano-scratching of SiGe alloy. The effect of scratching direction and Ge composition on material removal mechanism was discussed, aiming to understand the nanoscale deformation mechanism of SiGe alloy. The simulation results indicate that the machining direction and Ge composition have significant influences on the atomic flow and chip formation during nano-scratching. Besides, less subsurface damage and elastic recovery are observed when scratching along the (011)[100] direction with higher Ge composition. The highest crystal purity of the machined surface is achieved when scratching on the Si_60_Ge_40_ workpiece. Furthermore, the Ge composition has a significant influence on the workpiece temperature due to the variation of the thermal conductivity of the workpiece. This work could enrich the understanding of the deformation mechanism of SiGe alloy during nanoscale machining and open a potential to improve the machining performance of multicomponent semiconductor materials.

## Introduction

Silicon–germanium (SiGe) alloy is a new type of semiconductor material with any molar ratio of silicon (Si) and germanium (Ge). Like Si and Ge, their alloys could crystallize in the cubic-diamond structure whose lattice constant varies from 5.43 Å to 5.66 Å with an insignificant deviation from the Vegard’s law [1]. Due to its unique properties such as optical response and potential for bandgap, SiGe alloy is widely applied in thermoelectric devices [2], optoelectronic devices [3], infrared detectors [4] and semiconductor industry [5–7]. Since the surface morphology and subsurface defects could affect the material properties like photoelectric conversion efficiency [8, 9], high-performance devices usually require machined surface with nanoscale roughness and low machining damage. Therefore, exploration and understanding of the nanoscale machining technique is essential for high-quality manufacturing of these semiconductor components. However, owing to the inaccessibility of the nanoscale interaction, the deformation mechanism involved in nanoscale machining of SiGe alloy is difficult to be investigated experimentally.

In recent years, with the development of computer technique, molecular dynamics (MD) simulation has been widely accepted as a powerful method to explore the mechanism in nanoscale machining [10–13]. Based on the information of atomic interaction, MD method could handle the problem with large strain rate at atomic level. It has been successfully applied to investigate the nanoscale machining mechanism of semiconductor materials. For example, Cheng et al. [14] presented MD simulation to model the wear process of diamond tool during nano-scratching. They discussed the wear mechanism by calculation of the temperature and stress in the diamond tip. Fang et al. [15] investigated the nanoscale material removal mechanism of single-crystal Si by MD method. They proposed that the extrusion mechanism dominants the ductile mode removal process in nanoscale. Wang et al. [16] conducted MD simulation to investigate the crystal anisotropy of single-crystal Si during nanometric cutting. Their results indicate that the chip morphology and subsurface damage are greatly affected by the cutting plane and direction. Goel et al. [17] compared the structure evolution of single-crystal and polycrystal Si during nanometric cutting through MD simulation. It was indicated that the propensity for amorphization in single-crystal Si is significantly higher than that in the polycrystalline workpiece, signifying that grain boundaries eases the deformation process. Yan et al. [18] established a three-dimensional MD model to investigate the combination effect of the tool tip inclination and scratching direction on the nano-scratching process of single-crystal Si. Lai et al. [19] investigated the anisotropic subsurface deformation characters in nanometric cutting of single-crystal Ge based on MD simulation. In another research [20], they revealed that amorphous-damage-less and even amorphous-damage-free machined surface can be achieved by partially overlapped nano-cutting of Ge.

In general, SiGe alloys have three structural types: single crystalline, thin films, or polycrystalline. In the past two decades, the mechanical properties of SiGe alloy have been studied both experimentally and numerically. For instance, Wu et al. [21] studied nanomechanical behavior of the SiGe films with different annealed temperatures by nano-scratching and nano-indentation. Lin et al. [22] conducted nano-scratching experiment to explore the wear performance of SiGe epitaxial thin films. Chiang et al. [23] investigated the temperature-dependent mechanical properties of Si_80_Ge_20_ alloy via indentation experiment. Bathula et al. [24] studied the mechanical properties of Si_80_Ge_20_ alloy, which were synthesized employing spark plasma sintering. Recently, Pham and Fang [25] preformed MD simulation to explore the mechanical behavior of deposited Si_80_Ge_20_ thin film during nano-indentation and nano-scratching. Additionally, they studied the influences of grain size, alloy composition, and temperature effect on mechanical characters of polycrystalline SiGe though nano-indentation [26]. Their results indicate that the grain boundary has an apparent influence on the deformation mechanism in nano-scratching. However, in nanoscale machining, the material removal thickness ranges from tens to hundreds of nanometers, which is much smaller than the grain size. The material removal process mainly occurs in the single-crystal structure. Therefore, investigations of scratching process on single-crystal SiGe are important for improving the understanding of its machining mechanism in nanoscale.

In the present work, we performed MD simulation to explore the nano-scratching mechanism of single-crystal SiGe. The influences of Ge composition and workpiece orientation on material removal and subsurface damage formation were surveyed. Such information could be then used to analyze the nanoscale machining mechanism of SiGe alloy and open a potential to improve machining performance of multicomponent semiconductor materials. The simulation was conducted through the famous Large-scale Atomic/Molecular Massively Parallel Simulator (LAMMPS) [27] and OVITO [28] was employed to analyze the results.

## Simulation methods

### Modeling and simulation details

**Fig. 1:**
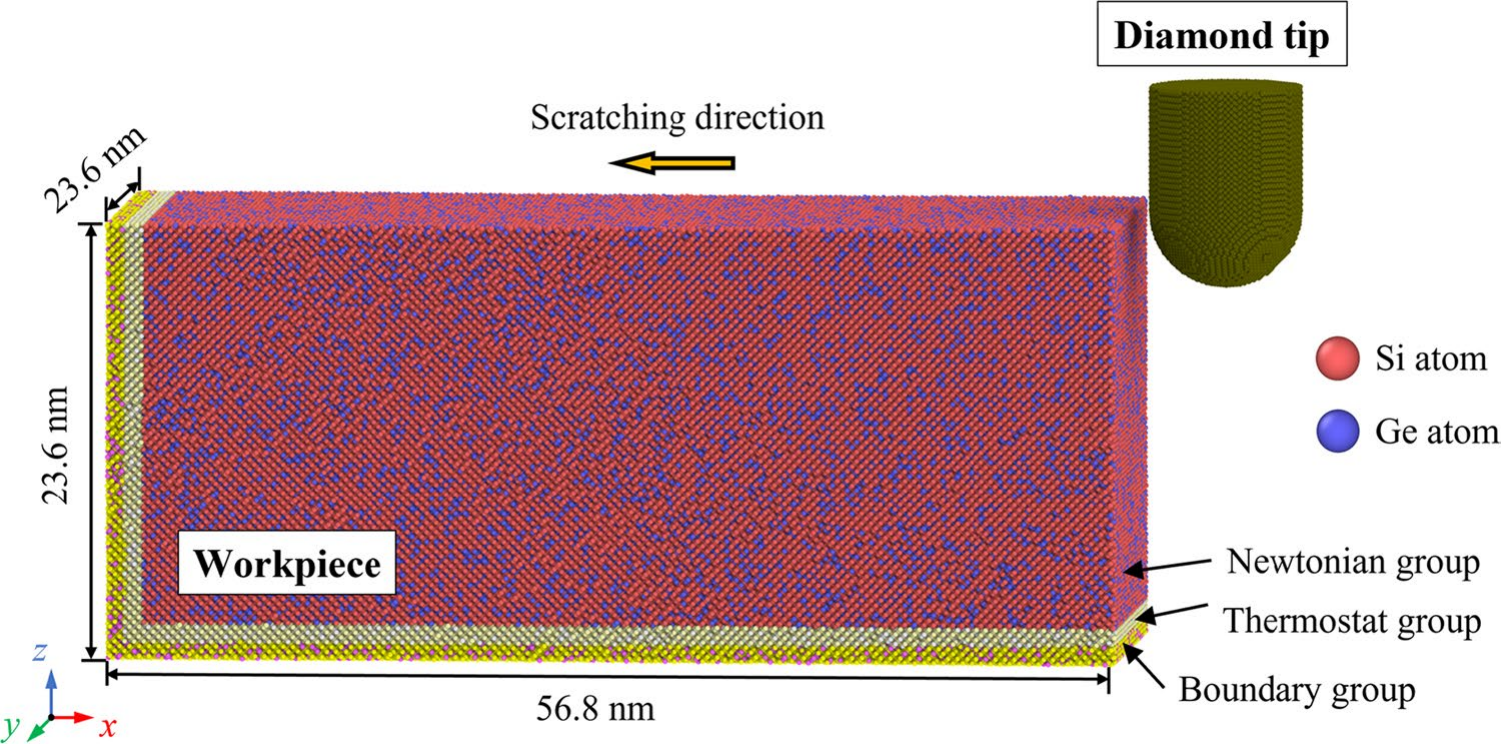
The MD model for nano-scratching of SiGe alloy

Figure 1 presents the nano-scratching model adopted in MD simulation. The SiGe workpiece is deformable while the diamond tip is set as a rigid body. The atoms in workpiece are divided into three groups: Newtonian group, thermostat group, and boundary group. During the scratching simulation, atoms in the Newton group follow the Newton’s second law of motion. Atoms in the thermostat group are maintained at room temperature to disperse the generated heat, while atoms in the boundary group are fixed in their balanced locations to support the workpiece. To simulate a bulk workpiece, periodic boundary condition (PBC) is used along the *y* direction. The workpiece is relaxed in the isothermal-isobaric (NPT) ensemble before scratching. Then the atoms in Newton group are scratched in the microcanonical (NVE) ensemble while the atoms in thermostat group are kept in the canonical (NVT) ensemble to rescale their temperature. Nano-scratching simulation is conducted on workpieces with various mole fractions of Ge. Meanwhile, to profoundly understand the effect of crystal anisotropy on the material removal process, the scratching direction is set as (001)[100], (011)[100] and (111)[110], respectively. Detail of the simulation parameters are listed in Table 1.

**Table 1:** Parameters of the MD model

Parameters	Value
Workpiece material	Si_80_Ge_20_, Si_60_Ge_40_, Si_40_Ge_60_, Si_20_Ge_80_
Workpiece dimensions	56.8 nm × 23.6 nm × 23.6 nm
Tip material	Diamond
Radius of the scratching tip	4 nm
Depth of scratching	4 nm
Scratching temperature	300 K
Scratching direction	(001)[100], (011)[100], (111)[110]
Scratching speed	50 m/s
Scratching distance	40 nm

### Potential function

Adopting a reliable potential function is critical for MD simulation to describe the atomic interactions. In this simulation, the Tersoff potential is used to characterize the interactions in workpiece atoms. It has been proven to describe the SiGe system accurately [26, 29] and widely used in simulations of SiGe superlattice [30], nanoporous SiGe [31], and Si/Ge nanotubes [32]. Depending on the Ge content, the lattice constants of Si_100−*x*_Ge_*x*_ alloy (*x*: mole fraction percent of Ge) can be fitted by [33]:

1 $$a\left( x \right) = 5.431 + 0.2\frac{x}{100} + 0.027\left( \frac{x}{100} \right)^{2}$$

**Fig. 2:**
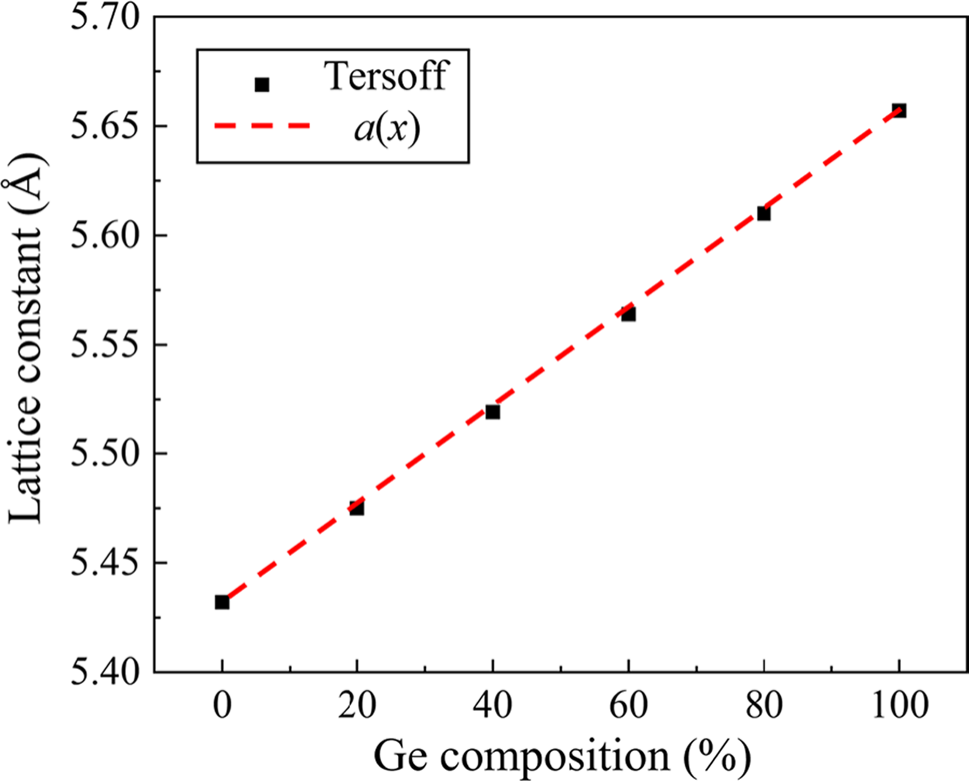
The calculated lattice constants of Si_100−*x*_Ge_*x*_ alloys by Tersoff potential and Eq. (1)

The calculated equilibrium lattice constants of Si_100−*x*_Ge_*x*_ by Tersoff potential are present in Fig. 2, which shows a good agreement with the fitting curve.

Furthermore, the interactions of C-Si and C-Ge are described by the Morse potential, which has been verified as an accurate and efficient selection [20, 34]. The Morse potential can be expressed as:

2 $$E\left( {r_{ij} } \right) = D_{{\text{M}}} [e^{{ - 2a(r_{ij} - r_{M} )}} - 2e^{{ - a(r_{ij} - r_{M} )}}]$$

where *D*_M_*, a,* and *r*_M_ represents the cohesion energy, elastic modulus, and equilibrium distance between atoms, respectively. Table 2 shows the parameters of the Morse potential adopted in this simulation.

**Table 2:** Parameters for Morse potential [20, 35]

Parameters	*D*_M_ (eV)	*a* (nm^−1^)	*r*_M_ (nm)
C-Si	0.435	46.487	0.19475
C-Ge	0.125778	25.8219	0.22324

## Results and discussion

### Material removal behavior

**Fig. 3:**
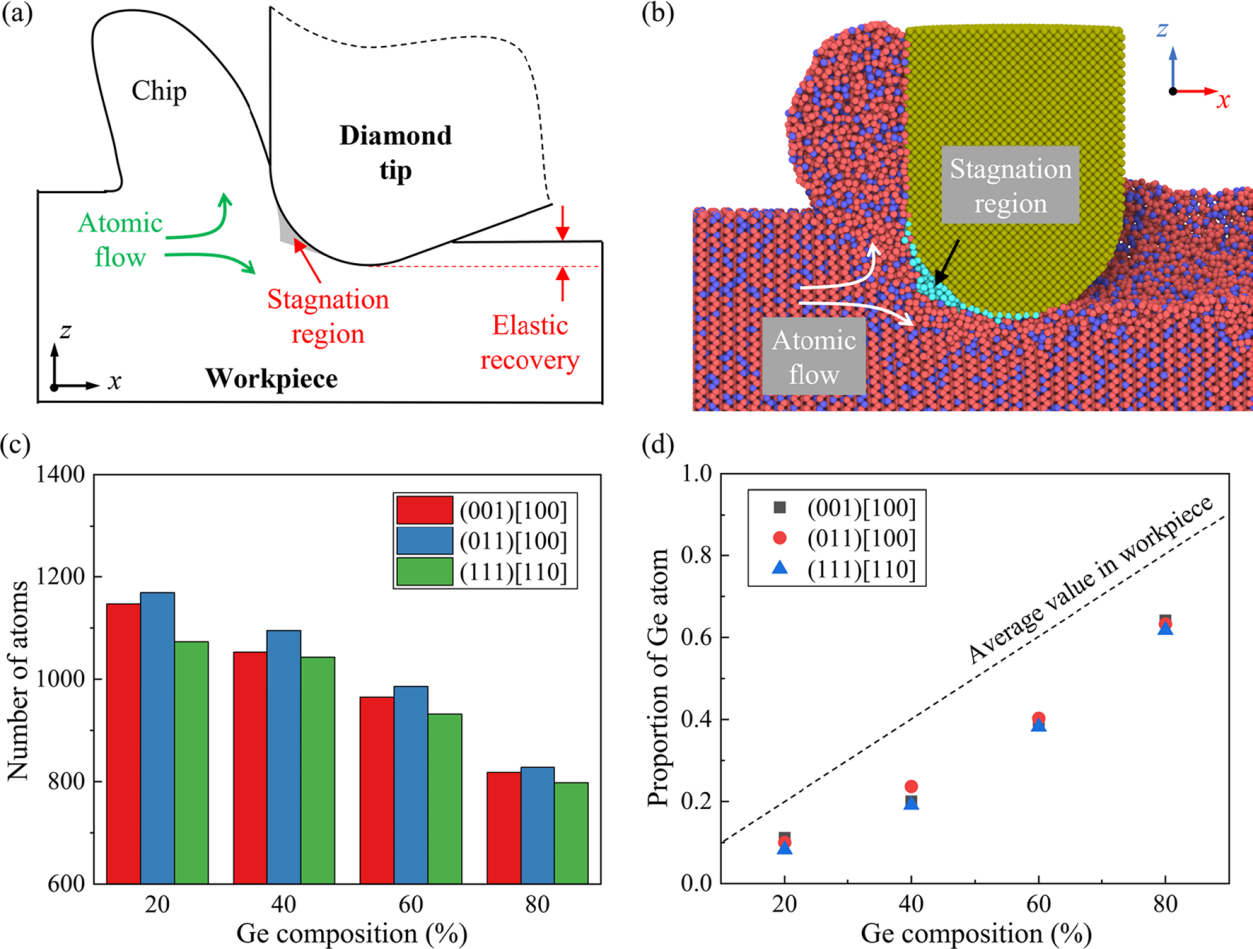
The stagnation region during nano-scratching: **a** Illustration of the stagnation region in the deformation region. **b** Snapshot of the stagnation region when machining along the (001) [100] direction on Si_80_Ge_20_ workpiece. **c** Number of the atoms in the stagnation region. **d** Proportion of Ge atoms in the stagnation region

In nano-scratching of brittle materials like single-crystal Si, workpiece atoms in the deformation region can be seperated into compressed material and chips by a stagnation region [36], as illustrated in Fig. 3a. Atoms in the stagnation region require more time and scratching distance to get rid of the confinement from the diamond tip. Figure 3b shows the snapshot of the stagnation region when machining on Si_80_Ge_20_ workpiece along the (001) [100] direction. The judgment condition for atoms in the stagnation region is that the relative atomic displacement (refer to the diamond tip) is less than one-tenth of that of the other workpiece atoms [37]. The number of atoms in stagnation region when the scratching distance reaches to 30 nm is present in Fig. 3c. It is observed that larger stagnation region is formed when scratching along the (011) [100] direction. Besides, smaller stagnation region can be formed with the increase in the Ge composition regardless the scratching direction. It is interesting that the content of Ge atom in the stagnation region in all cases are apparently less than the average value in workpiece, as shown in Fig. 3d. Note that since the Si–Si bonding is stronger than Ge–Ge bonding [38], less energy is required to destroy the crystal structure of SiGe with higher Ge composition. The plastic deformation and atomic flow would be facilitated when the Ge composition is increased, leading to smaller stagnation region. Meanwhile, the Si clusters are more stable and tend to concentrate in the stagnation region, which decreases the proportion of Ge atoms. Therefore, during the scratching process, the mechanical properties of material in the deformation region can be different with the whole workpiece due to the concentration of Si atoms.

**Fig. 4:**
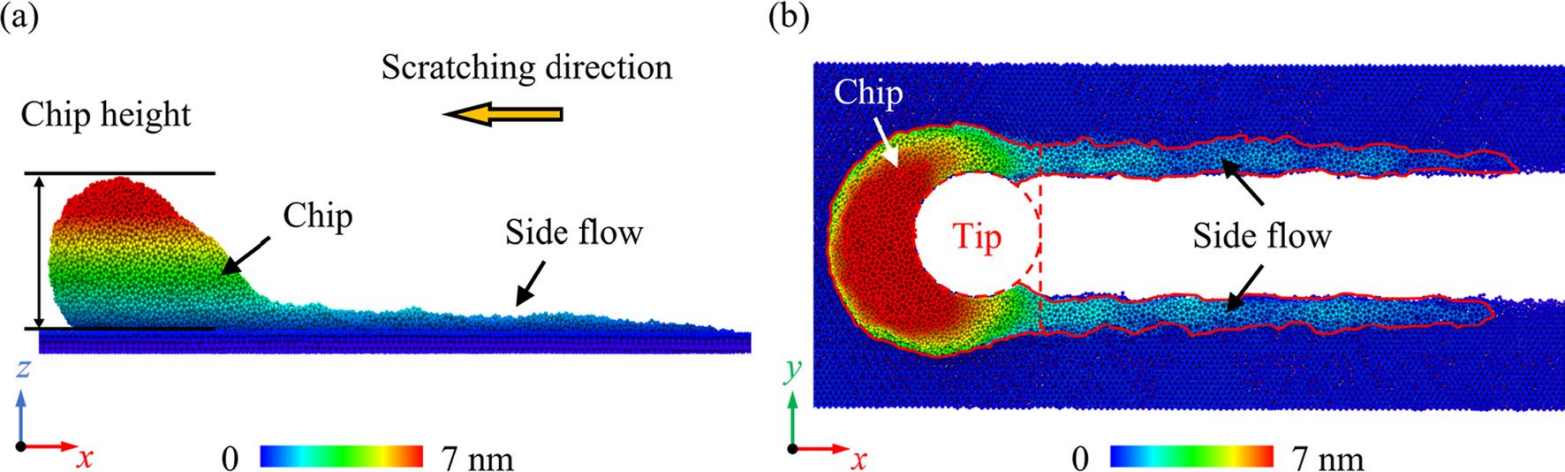
The chip morphology when machining along the (001) [100] direction on Si_80_Ge_20_ workpiece as the scratching distance reaches to 40 nm: **a** the front view and **b** the top view

**Fig. 5:**
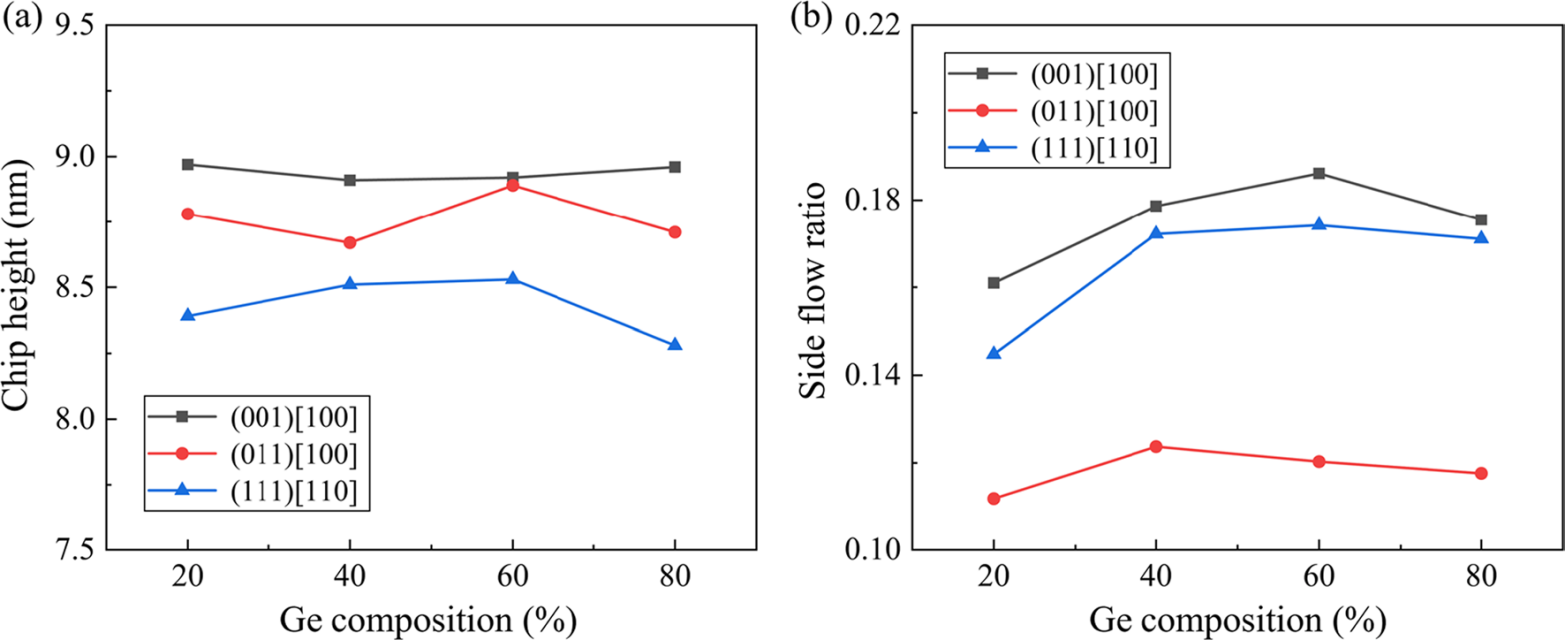
Variation of the **a** chip height and **b** side flow ratio as a function of the scratching direction and Ge composition when the scratching distance reaches to 40 nm

During the nano-scratching process, atoms above the stagnation region would be piled up on the uncut surface to form chips, as shown in Fig. 4. In addition, a part of these atoms can move along the *y*-direction and flow to the side of the tool edge, which is identified as the chip side flow [39, 40]. These atoms would be left on the uncut surface and have a great influence on the machined surface roughness [37]. In the present work, the side flow atoms are identified as those above the uncut surface and left behind the scratching tip, as shown in Fig. 4b. To illustrate the material removal behavior quantitatively, the chip height with different Ge composition and scratching direction is calculated, as shown in Fig. 5a. It is observed that the Ge composition has an inapparent influence on the chip height while the effect of scratching direction is much more obvious. Similar with single-crystal Si, less atoms were piled up to form chips when scratching on the (111) crystal plane [41]. Figure 5b presents the variation of side flow ratio as a function of the Ge composition and scratching direction, which is defined as the ratio between number of the side flow atoms and total piled-up atoms. It is found that the side flow ratio is much smaller when scratching along the (011) [100] direction, which indicate that less material is left on the uncut surface during scratching. Meanwhile, with increasing Ge composition, side flow ratio raises firstly then shows slight decrease regardless the scratching direction. As mentioned above, the atomic flow in the deformation region can be promoted with an increase in the Ge composition, which is advantageous for side flow of workpiece material. On the other hand, it has been reported that the side flow is facilitated when the machining temperature and internal stress in the deformation zone are increased [42]. As discussed below, the internal stress and temperature in workpiece are gradually decreased as the Ge composition raises from 40 to 80%. Therefore, upon the interaction of these factors, the maximum side flow ratio is observed in Si_60_Ge_40_ or Si_40_Ge_60_ regarding the scratching direction.

### Machined surface morphology

**Fig. 6:**
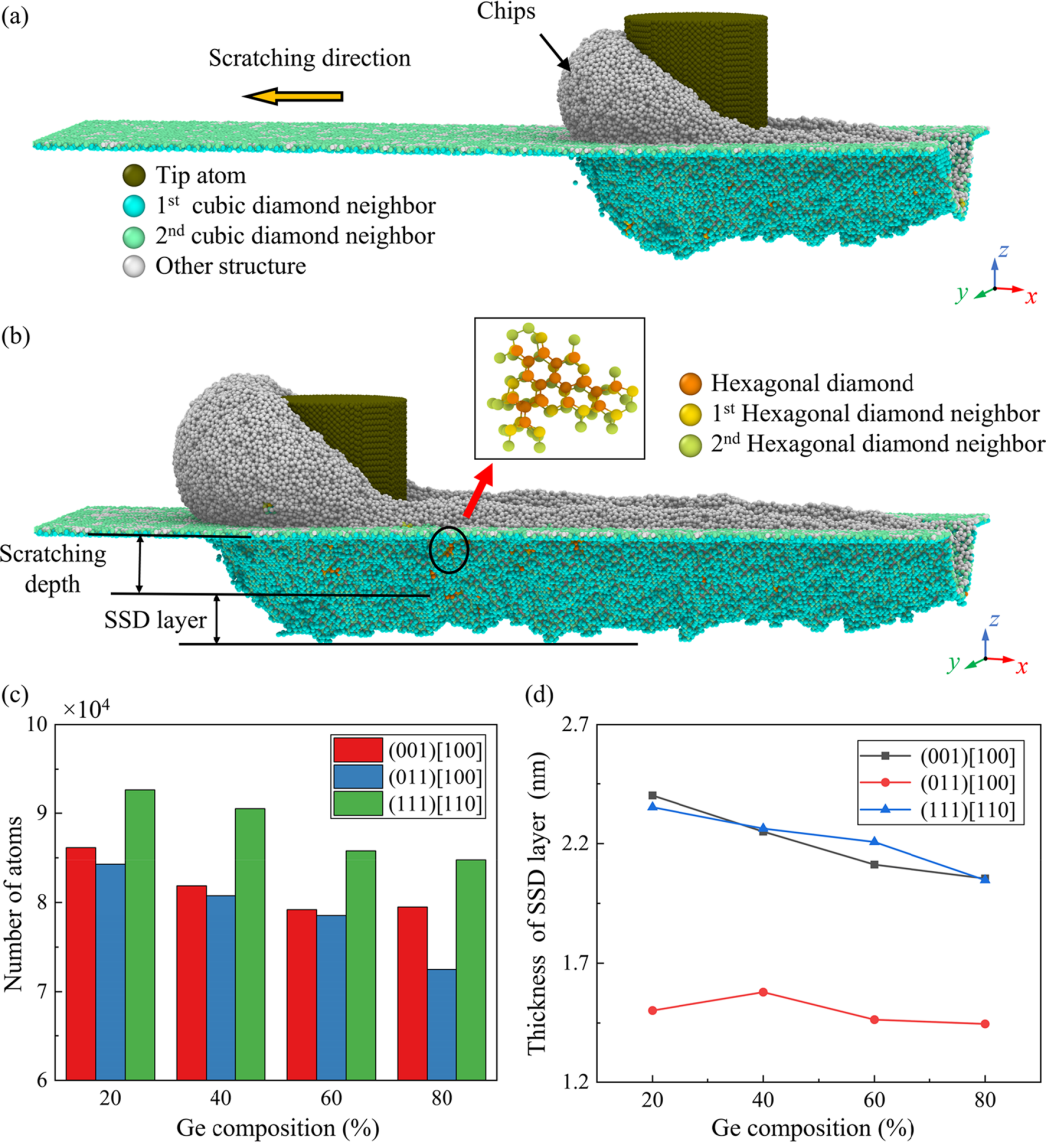
Formation of subsurface damage during nano-scratching: **a** and **b** Illustration of the subsurface damage (SSD) when machining along the (001) [100] direction on Si_80_Ge_20_ workpiece at a scratching distance of 20 nm and 40 nm. **c** Number of the atoms in other structure. **d** Variation of the thickness of the SSD layer at a scratching distance of 40 nm

After the diamond tip passes the scratching area, subsurface damage (SSD) can be generated beneath the machined surface. Figure 6a, b demonstrates the scratching-induced SSD where the crystal structure of workpiece is identified by the Common Neighbor Analysis (CNA) [43]. The structure of workpiece atoms is classified as cubic diamond structure, hexagonal diamond structure, and other structure, which mainly contains metallic phase, amorphous phase, and other defective structures [44, 45]. The SSD region can be determined by identifying the neighbors of the cubic diamond atoms. Figure 6c shows the number of workpiece atoms that are identified as other structure at a scratching distance of 40 nm. It is observed that fewest defective atoms are generated when scratching along the (011)[100] direction. Meanwhile, the number of the generated defective atoms is apparently decreased as the increase in Ge composition. Figure 6d shows the variation of the average thickness of the SSD layer. It is concluded that better subsurface quality can be obtained when scratching along the (011)[100] direction. Besides, the thickness of the SSD layer is gradually decreased as the Ge composition increases. This variation can be attributed to the larger atomic interval in workpiece and less released strain energy during scratching, which reduces the compression near the tip edge and suppresses the formation of SSD.

**Fig. 7:**
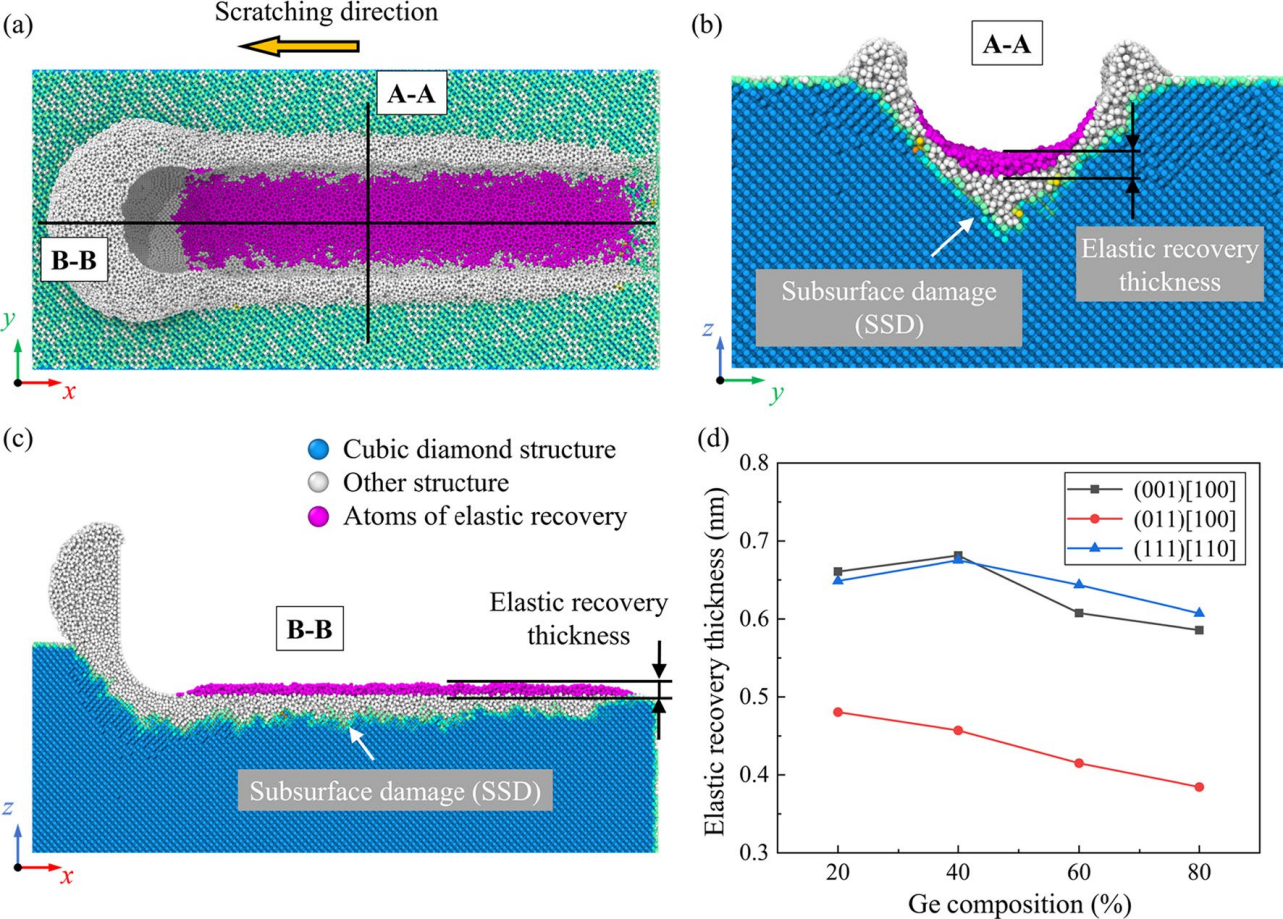
Elastic recovery on the machined surface: **a**–**c** snapshots of the elastic recovery layer when machining along the (001) [100] direction on Si_80_Ge_20_ workpiece. **d** The variation of the elastic recovery thickness

During the nano-scratching process, the highly compressed atoms beneath the stagnation region tend to restore their equilibrium positions to relieve the residual stresses after scratching. For semiconductor material like Si, back transition from these high-compressed phases is usually accompanied with an increase in volume [45]. This phenomenon can be recognized as the elastic recovery [46] or swelling effect [47], which has a great influence on the machined surface morphology. Figure 7a–c presents the snapshots of the elastic recovery layer when scratching on the Si_80_Ge_20_ workpiece along the (001)[100] direction. It is observed that elastic recovery is most obvious near the center of the tip edge and gradually decreases to zero at two sides of the scratching trace. Figure 7d shows the average elastic recovery thickness (defined in Fig. 7b) as the scratching distance reaches to 40 nm. It is observed that the minimum elastic recovery thickness is obtained when scratching along the (011)[100] direction. Besides, since the compression of atoms in the deformation region is decreased with increasing Ge composition, the average elastic recovery thickness is gradually decreased as the Ge composition raises, especially for the (011)[100] direction.

### Phase transition

**Fig. 8:**
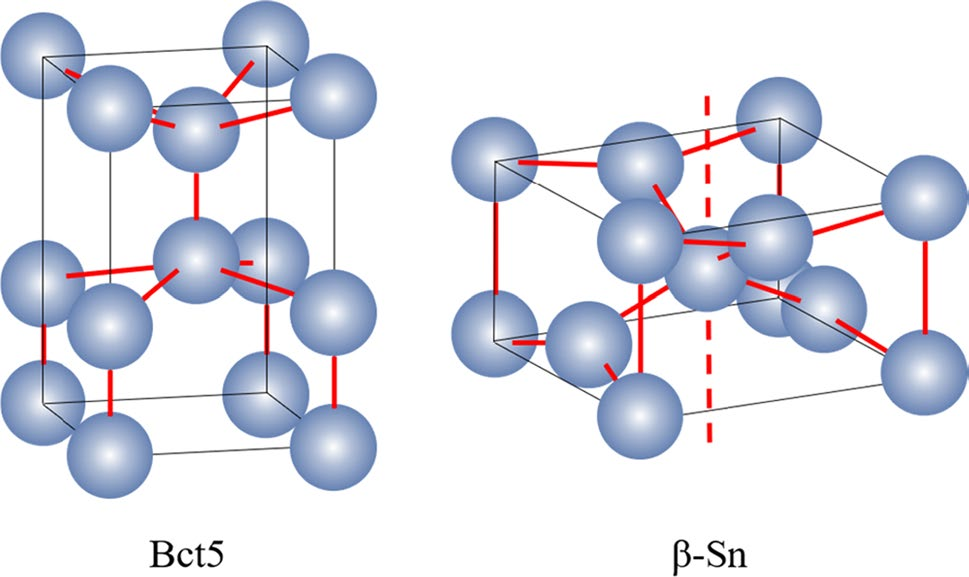
Illustration of the Bct5 and β-Sn structure

**Fig. 9:**
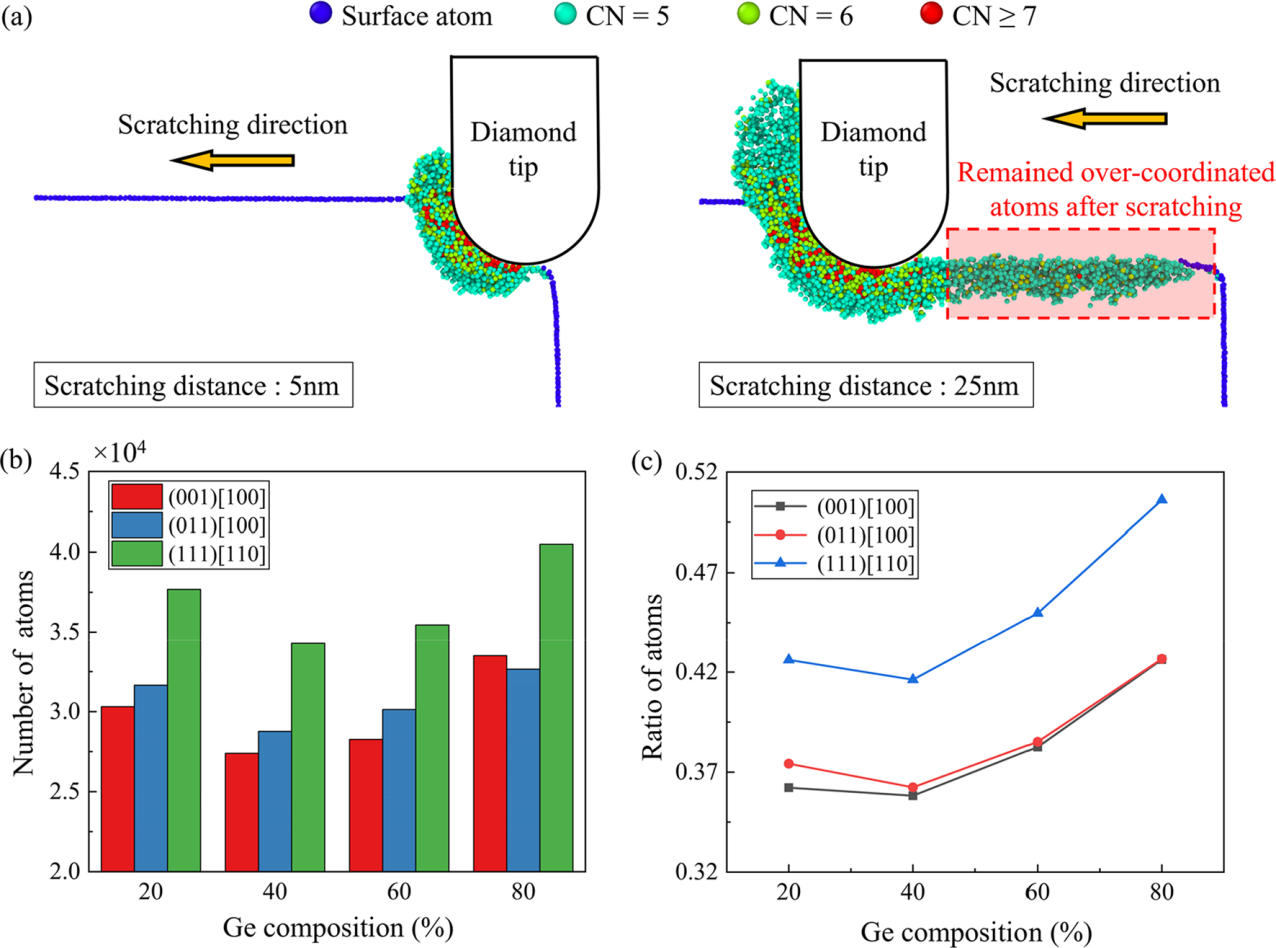
The over-coordinated atoms during nano-scratching: **a** Distribution of the over-coordinated atoms when machining along the (001) [100] direction on Si_80_Ge_20_ workpiece as the tip advances of 5 nm and 25 nm. **b** Number of the over-coordinated atoms at a scratching distance of 40 nm. **c** Ratio of the remained over-coordinated atoms to the total over-coordinated atoms

In nano-scratching of single-crystal Si and Ge, phase transition into structures with high coordination number (CN) like Bct5 (CN = 5) and β-Sn (CN = 6) phases (as illustrated in Fig. 8) can be detected in the deformation region, which is an important source of ductile mode removal [20, 48]. Figure 9a are the snapshots showing the distribution of the over-coordinated atoms with the progress of scratching along the (001)[100] direction on Si_80_Ge_20_ workpiece. As the scratching tip proceeds, the deformation layer expands ahead the scratching direction accompanied with the generation of the over-coordinated atoms. Most atoms with large coordination number (CN ≥ 6) are observed near the tip edge while considerable 5-coordinated atoms are left on the machined surface after scratching. Figure 9b shows the number of over-coordinated atoms at a scratching distance of 40 nm. It is observed that more over-coordinated atoms are generated when scratching along the (111)[110] direction. Furthermore, the ratio of the remained over-coordinated atoms to the total over-coordinated atoms is calculated to measure the machined surface quality, as shown in Fig. 9c. It is observed that more over-coordinated atoms tend to remain on the machined surface when scratching along the (111)[110] direction. Meanwhile, regardless the scratching direction, fewest over-coordinated atoms are left on the Si_60_Ge_40_ workpiece, which implies highest crystal purity of the machined surface.

**Fig. 10:**
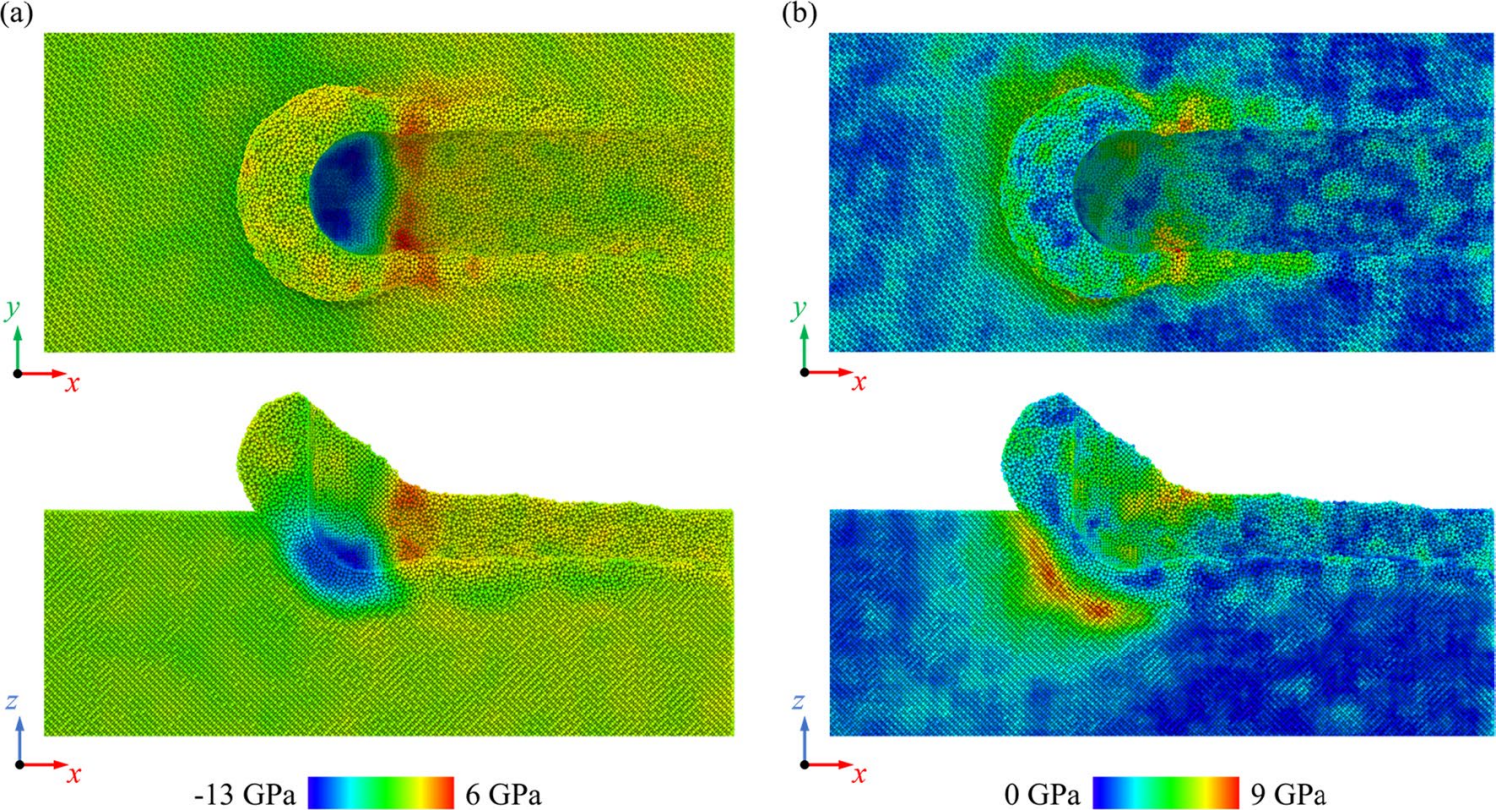
The stress distribution when machining along the (001) [100] direction on Si_80_Ge_20_ workpiece, which is calculated by the stress tensors in LAMMPS [50, 51]: **a** Hydrostatic stress distribution and **b** Von Mises distribution

**Fig. 11:**
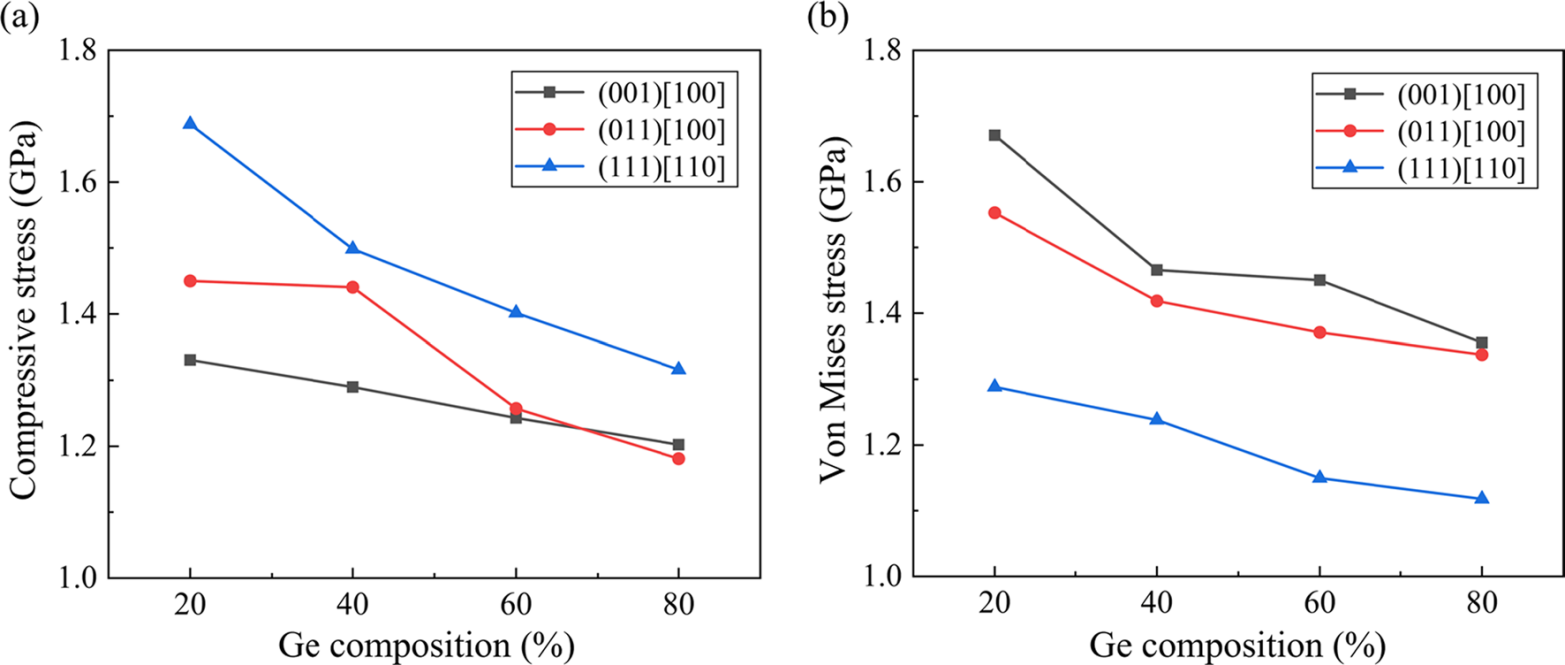
Variation of the **a** average compressive stress and **b** average von Mises stress in workpiece during scratching as a function of the scratching direction and Ge composition

Internal stress is an important factor that affect the phase transition in nano-scratching. Figure 10 presents the distribution of the hydrostatic stress and von Mises stress when machining along the (001) [100] direction on Si_80_Ge_20_ workpiece at a scratching distance of 25 nm. The highly compressed region mainly concentrates near the tip edge while obvious tensile stress is observed behind the scratching tip, indicating tearing of the surface material by the tool movement. Meanwhile, as presented in Fig. 10b, the region with high von Mises stress mainly locates in the subsurface workpiece beneath the diamond tip, implying the shear deformation in workpiece during scratching. Figure 11 shows the average compressive stress and von Mises stress of workpiece during scratching. It is observed that the average stress decreases obviously with increasing Ge composition regardless the scratching direction, which is disadvantageous for the high-pressure phase transition. However, it is reported that for SiGe alloy, the critical pressure *P*_T_ for the transition from cubic diamond phase to β-Sn phase decreases with increasing Ge composition [49]. Therefore, during the nano-scratching process, the formation of the over-coordinated atoms decreases firstly and then increases as the Ge composition increases, and fewest over-coordinated atoms are generated when scratching the Si_60_Ge_40_ workpiece, as shown in Fig. 9c.

### Scratching forces & temperature

**Fig. 12:**
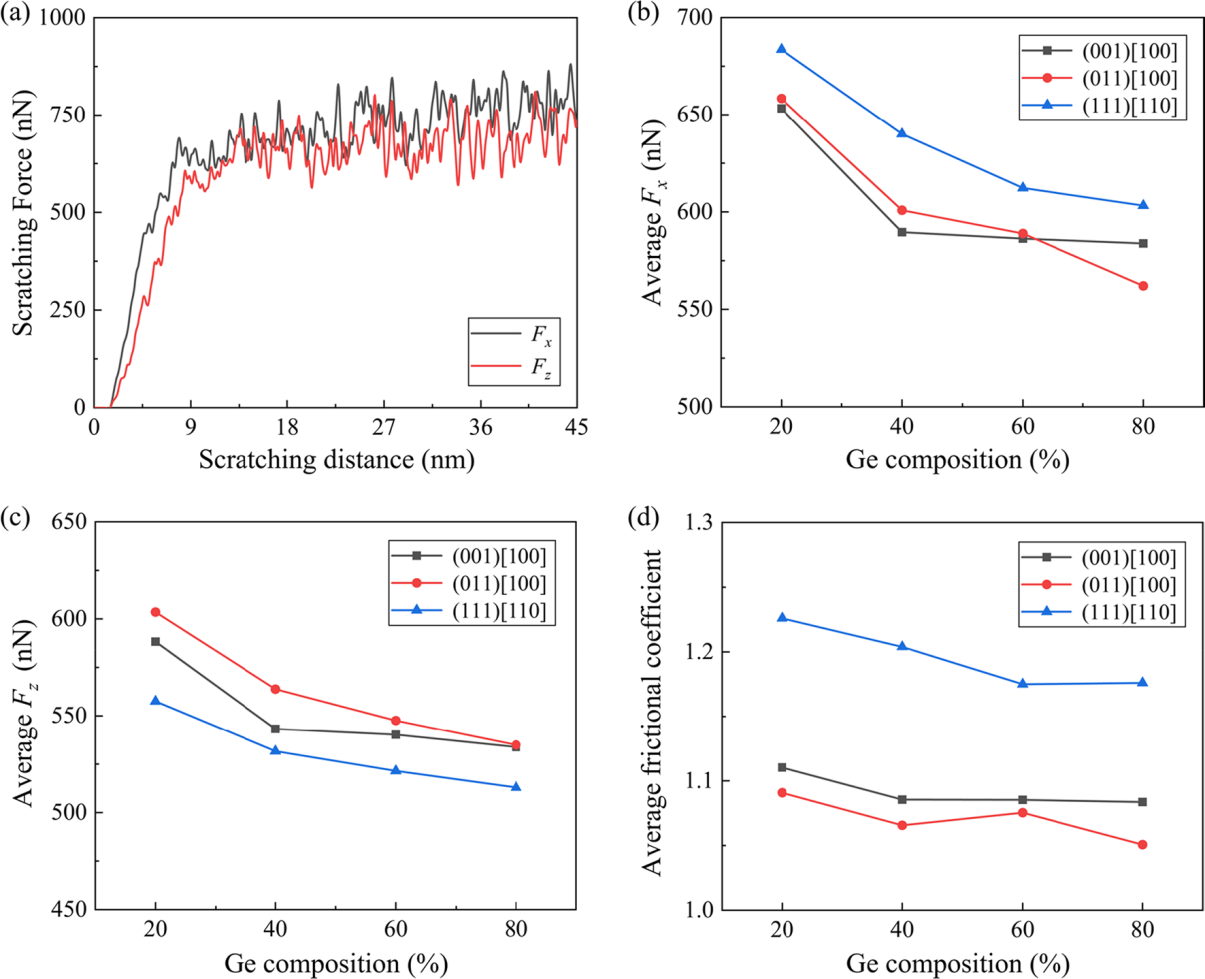
**a** The transient scratching forces when machining along the (001) [100] direction on Si_80_Ge_20_ workpiece. **b** and **c** the average tangential and normal forces. **d** The average frictional coefficient

Figure 12a shows the transient scratching forces in the tangential (*x*) and normal (*z*) directions when machining along the (001) [100] direction on Si_80_Ge_20_ workpiece. A sharp increase in the scratching forces is observed in the initial stage due to the change of the contact area between diamond tip and workpiece. Subsequently, the transient scratching forces fluctuate around at constant values in a relatively steady stage. Figure 12b, c presents the average forces in the steady stage with different scratching directions and Ge compositions. It is observed that the average tangential force and normal force both decrease obviously with increasing Ge composition as the hardness of workpiece is decreased. Furthermore, the average frictional coefficient is calculated based on the scratching forces in the steady scratching stage, as shown in Fig. 12d. The maximum frictional coefficient is observed when scratching along the (111)[110] direction, which is similar with previous result when machining single-crystal Si [52]. A notable observation is that the frictional coefficient decreases slightly with increasing Ge composition regardless the scratching direction. This variation can be attributes to the stronger adhesion between atoms in workpiece and diamond tip since the bonding energy for Si–C (318 kJ/mol) is larger than Ge-C (238 kJ/mol) [38].

**Fig. 13:**
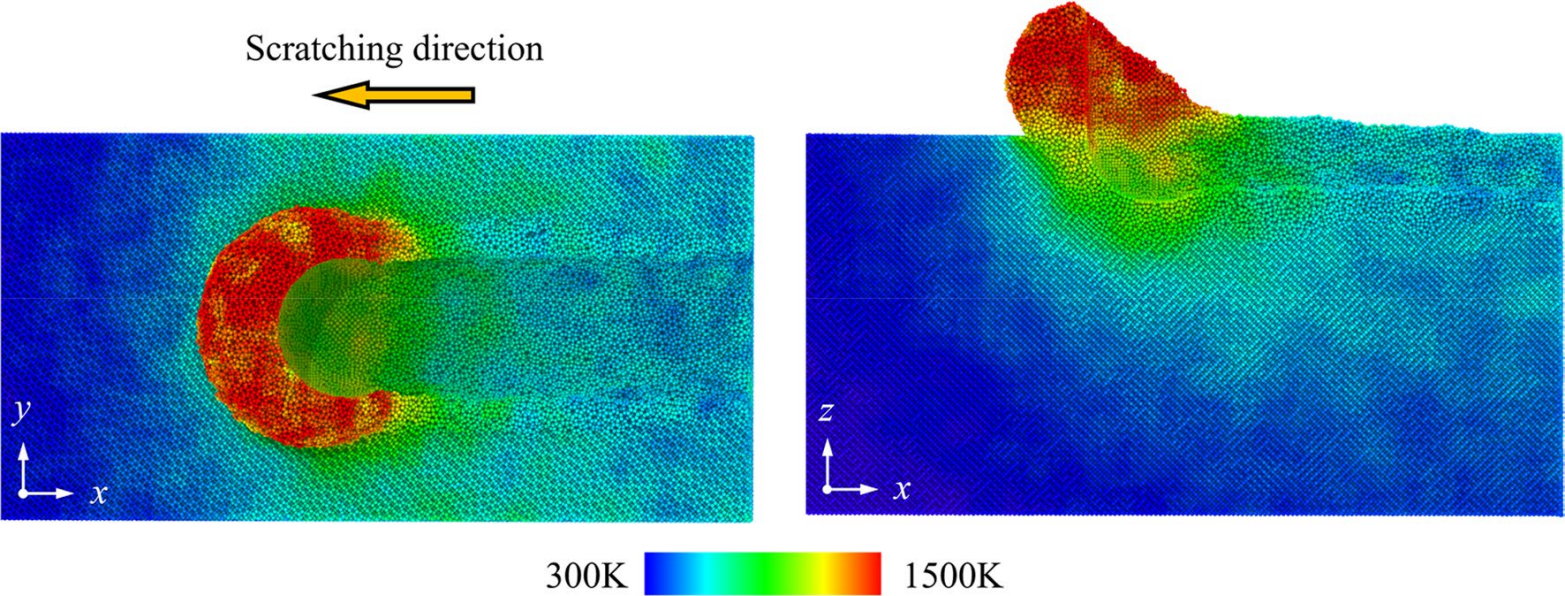
Temperature distribution when scratching on the Si_80_Ge_20_ workpiece along the (001)[100] direction as the scratching distance reaches to 25 nm

**Fig. 14:**
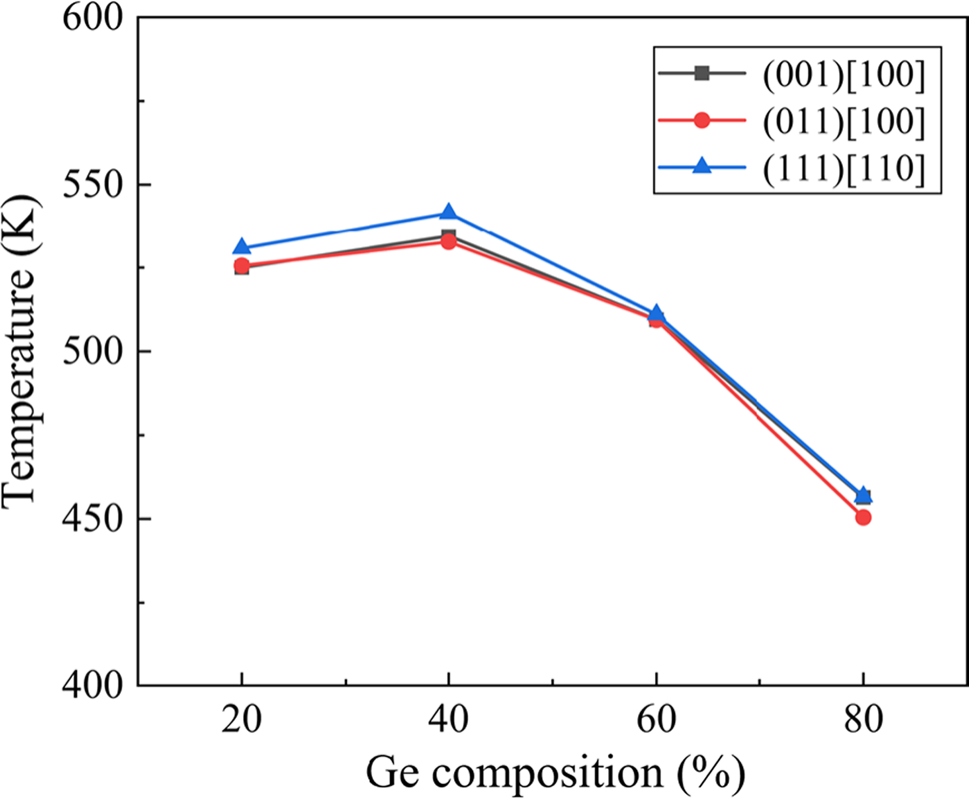
Average workpiece temperature as a function of scratching direction and Ge composition

Figure 13 shows the temperature distribution when scratching along the (001) [100] direction on Si_80_Ge_20_ workpiece. It is observed that the highest temperature of about 1500 K is produced in the chip around the scratching tip. This is due to the energy accumulation from the friction at the chip-tip interface and plastic deformation occurred in workpiece. Furthermore, Fig. 14 shows the average workpiece temperature when the scratching distance reaches to 40 nm. It can be concluded that the scratching direction has an inapparent influence on the workpiece temperature while with the increase in the Ge composition, the workpiece temperature increases firstly and then decreases obviously. Since the energy of Ge–Ge bonding is smaller than Si–Si bonding, less energy is generated during scratching with increasing Ge composition. However, as the Ge composition rises from 0 to 100, the thermal conductivity of SiGe alloy decreases firstly and then increasing rapidly [53, 54], causing obvious difference in energy dissipation. Therefore, the workpiece temperature can be influenced and the behavior of atomic flow and dislocation movement is affected, which are important factors in determining the surface morphology and plastic deformability [55, 56].

## Conclusions

In the present work, molecular dynamics simulation was conducted to investigate the nano-scratching mechanism of SiGe alloy. Effects of the scratching direction and Ge composition on material removal mechanism and SSD formation were discussed. During the scratching process, larger stagnation region can be formed when scratching along the (011)[100] direction with smaller Ge composition. Meanwhile, concentration of Si atoms is observed in the stagnation region. Besides, strongest side flow is observed when scratching on Si_60_Ge_40_ or Si_40_Ge_60_ workpiece regarding the scratching direction. Furthermore, smaller SSD layer is formed when scratching along the (011)[100] direction with higher Ge composition. On the machined surface, fewest over-coordinated atoms are left on the Si_60_Ge_40_ workpiece regardless the scratching direction, which implies highest crystal purity of the machined surface. Moreover, the average tangential force and normal force both decrease obviously with increasing Ge composition. While the maximum frictional coefficient is observed when scratching along the (111)[110] direction on workpiece with smaller Ge composition. In addition, the Ge composition has a significant influence on the workpiece temperature due to the variation of the thermal conductivity. These results could enrich the understanding on the deformation mechanism of SiGe alloy in nanoscale machining and open a potential to improve the machining performance of multicomponent semiconductor materials.

## Data Availability

Data underlying the results presented in this paper are not publicly available at this time but may be obtained from the authors upon reasonable request.
